# Ketone monoester attenuates oxygen desaturation during weighted ruck exercise under acute hypoxic exposure but does not impact cognitive performance

**DOI:** 10.1113/EP091789

**Published:** 2024-08-27

**Authors:** Tyler S. McClure, Jeffrey Phillips, Dawn Kernagis, Kody Coleman, Ed Chappe, Gary R. Cutter, Brendan Egan, Todd Norell, Brianna J. Stubbs, Marcas M. Bamman, Andrew P. Koutnik

**Affiliations:** ^1^ School of Health and Human Performance Dublin City University Dublin Ireland; ^2^ Healthspan, Resilience and Performance Research Florida Institute for Human and Machine Cognition Pensacola Florida USA; ^3^ Department of Neurosurgery University of North Carolina Chapel Hill North Carolina USA; ^4^ Buck Institute for Research on Aging Novato California USA; ^5^ Sansum Diabetes Research Institute Santa Barbara California USA

**Keywords:** β‐hydroxybutyrate, cognitive performance, exogenous ketones, heart rate variability, oxygen saturation

## Abstract

Acute ingestion of exogenous ketone supplements in the form of a (*R*)‐3‐hydroxybutyl (*R*)‐3‐hydroxybutyrate (R‐BD R‐βHB) ketone monoester (KME) can attenuate declines in oxygen availability during hypoxic exposure and might impact cognitive performance at rest and in response to moderate‐intensity exercise. In a single‐blind randomized crossover design, 16 males performed assessments of cognitive performance before and during hypoxic exposure with moderate exercise [2 × 20 min weighted ruck (∼22 kg) at 3.2 km/h at 10% incline] in a normobaric altitude chamber (4572 m, 11.8% O_2_). The R‐BD R‐βHB KME (573 mg/kg) or a calorie‐ and taste‐matched placebo (∼50 g maltodextrin) were co‐ingested with 40 g of dextrose before exposure to hypoxia. The R‐βHB concentrations were rapidly elevated and sustained (>3 mM; *P *< 0.001) by KME. The decline in oxygen saturation during hypoxic exposure was attenuated in KME conditions by 2.4%–4.2% (*P *< 0.05) compared with placebo. Outcomes of cognitive performance tasks, in the form of the Defense Automated Neurobehavioral Assessment (DANA) code substitution task, the Stroop color and word task, and a shooting simulation, did not differ between trials before and during hypoxic exposure. These data suggest that the acute exogenous ketosis induced by KME ingestion can attenuate declining blood oxygen saturation during acute hypoxic exposure both at rest and during moderate‐intensity exercise, but this did not translate into differences in cognitive performance before or after exercise in the conditions investigated.

## Abstract (highlights)

- **What is the central question of this study?**
  Can exogenous ketosis act as a countermeasure to declines in blood oxygen saturation and cognitive performance during acute hypoxic exposure while performing a weighted ruck exercise?
- **What is the main finding and its importance?**
  Acute exogenous ketosis via ingestion of a drink containing the (*R*)‐3‐hydroxybutyl (*R*)‐3‐hydroxybutyrate ketone monoester prior to acute hypoxic exposure attenuated hypoxia‐induced declines in blood oxygen saturation but had no effect on cognitive performance during exercise.

## 1 INTRODUCTION

Hypoxia occurs when oxygen availability does not match the demand required to maintain homeostasis, most often owing to insufficient oxygen delivery to a tissue because of reduced blood oxygen saturation ($S_{pO_{2}}$) or reduced tissue perfusion (Sarkar et al., 2017). During exposure to high altitude, the partial pressure of oxygen declines and reduces the driving force for the diffusion of oxygen across alveolar membranes, which decreases $S_{pO_{2}}$. One consequence can be acute mountain sickness (AMS), which predominately includes symptoms of headache, weakness, fatigue, nausea, insomnia and decreased appetite (Peacock, 1998). These result from local tissue hypoxia with decreases in oxidative capacity and cerebral metabolic rate (Williams et al., 2019). Blunted oxidative capacity during hypoxia impairs physical performance because relative workload intensities are higher (Deb et al., 2018). As the severity of hypoxia increases, reliance on anaerobic metabolism rises, elevating the relative difficulty of performing and maintaining performance in a given physical task (Deb et al., 2018). Moreover, reduced cerebral metabolic rate during hypoxic exposure is detrimental to cognitive performance by negatively impacting working memory (Legg et al., 2016; Malle et al., 2013), cognitive flexibility (Asmaro et al., 2013; Turner et al., 2015), attention (Asmaro et al., 2013; Stepanek et al., 2014; Turner et al., 2015), executive function (Turner et al., 2015) and auditory processing (Beer et al., 2017).

Military personnel are often required simultaneously to perform high‐level cognitive and physical tasks in hypoxic environments without a prior period of adaptation, either acute or chronic, to hypobaric hypoxia. Therefore, any therapeutic strategy to mitigate the effects of hypoxic exposure might need to consider potential interaction effects of physical work or exercise and whether this influences countermeasure utility. Acute bouts of moderate‐intensity exercise lasting ≥20 min have demonstrated the ability to improve cognitive performance during hypoxic exposure (Chang et al., 2012). The cardiorespiratory and metabolic demands of exercise enhance the ability to maintain a higher level of cerebral metabolic rate, therefore mitigating the negative consequences of acute hypoxic exposure (Ando et al., 2020). These ergogenic effects are likely to result from increases in cerebral blood flow, catecholamine concentrations (dopamine, adrenaline and noradrenaline) and brain‐derived neurotrophic factor (Chang et al., 2012; McMorris et al., 2017, 2016). However, exercise as a countermeasure to combat hypoxic exposure is effective only during exercise, thereby requiring sustained moderate‐intensity exercise, which limits its practical use when acute hypoxia exposure is prolonged.

Attenuating the decline in $S_{pO_{2}}$ is the most promising countermeasure for mitigating the negative consequences of acute hypoxic exposure by maintaining cerebral metabolic rate and a higher rate of aerobic metabolism during exercise (Ando et al., 2020; Scott et al., 2016). Acetazolamide is the gold standard of treatment for AMS, primarily by increasing $S_{pO_{2}}$ and reducing symptoms of hypoxic exposure, but in comparison to placebo during hypoxic exposure, acetazolamide can impair exercise capacity (Bradwell et al., 2018) and neuropsychological measures of concentration, cognitive processing speed, reaction time, short‐term memory and working memory (Wang et al., 2013). Therefore, a hypoxia countermeasure is needed that mitigates declines in $S_{pO_{2}}$, decreases symptoms of AMS and maintains or attenuates declines in physical and/or cognitive performance during exercise or other physically demanding tasks.

Exogenous ketone supplements, particularly in the form of ketone esters, are an emerging therapeutic modality that might counter hypoxia (Evans et al., 2022; Poffé, Robberechts et al., 2021). Ingestion of exogenous ketone supplements elevates circulating concentrations of the ketone bodies β‐hydroxybutyrate (βHB) and acetoacetate (AcAc) within minutes of ingestion, and these increases can be maintained for several hours (Stubbs et al., 2017; Vandoorne et al., 2017), in comparison to dietary ketosis, which needs a multiple‐week adaptation period to achieve similar benefits (Burke, 2021). This acute transient increase has been termed ‘acute nutritional ketosis’ or ‘exogenous ketosis’ and has been observed consistently to have effects on metabolism, both at rest and during and after exercise (Evans et al., 2022). Acute ingestion of a ketone monoester (KME) in the form of (*R*)‐3‐hydroxybutyl (*R*)‐3‐hydroxybutyrate (R‐BD R‐βHB) has been shown to attenuate declines in cognitive performance in hypoxic conditions at rest (Coleman et al., 2021). Both at rest (Coleman et al., 2021) and during exercise (Poffé et al., 2021; Prins et al., 2021), acute ingestion of KME attenuates the decline in $S_{pO_{2}}$ in hypoxic conditions. Indeed, there might be overlapping mechanisms of action for acute nutritional ketosis and exercise to improve cognition during hypoxic exposure (Prins et al., 2021). Therefore, the aim of the present study was to investigate whether acute ingestion of R‐BD R‐βHB KME impacts $S_{pO_{2}}$ and cognitive performance in young, healthy males experiencing acute hypoxic exposure (simulated 15,000 ft/4572 m, normobaric, 11.8% O_2_) at rest and during moderate‐ to vigorous‐intensity exercise in the form of a weighted treadmill ruck. We hypothesized that the acute ingestion of R‐BD R‐βHB KME would attenuate the declines in $S_{pO_{2}}$ and cognitive performance caused by acute hypoxic exposure.

## 2 MATERIALS AND METHODS

### 2.1 Ethical approval

Each participant provided written informed consent to participate after written and verbal explanations of the procedures, in accordance with the protocol approved by the Florida Institute for Human & Machine Cognition Institutional Review Board (IRB‐2020‐0005) and Office of Human Research Oversight, US Army Medical Research and Development Command, and followed DoD Instruction 3216.02, ‘Protection of Human Subjects and Adherence to Ethical Standards in DoD‐Conducted and Supported Research’ (E02621.1a). The study conformed to the standards set by the *Declaration of Helsinki*, except for registration in a trials database.

### 2.2 Participants

Sixteen healthy males (1.81 ± 0.06 m, 82.0 ± 8.3 kg and 24.4 ± 4.3 years of age) provided written informed consent to participate after written and verbal explanations of the procedures and completed all components of the study. An a priori sample size calculation was performed based on two conditions with up to eight serial measurements in a repeated‐measures within‐between interaction design. To detect an effect size, *f*, of 0.253 [based on a partial eta squared (η^2^_p_) = 0.06; ‘moderate’ effect] for a given parameter at a type I error rate (α) of 0.05 and a power (1 − β) of 0.8 required a sample size of *n *= 16 (G*Power v.3.1).

Inclusion criteria were as follows: (1) exercised 150 min per week at moderate to high intensity for ≥1 year; (2) 18–35 years of age; and (3) consumed a standard American diet (Snetselaar et al., 2021). Exclusion criteria were as follows: (1) history of smoking; (2) metabolic or cardiovascular disease; (3) orthopaedic, musculoskeletal, neurological or psychiatric disorder and/or any medical conditions that prohibit exercise; (4) habitual prescription medications; (5) exogenous ketone supplements or following a low‐carbohydrate or ketogenic diet (Volek et al., 2024); or (6) use of ergogenic aids affecting cognitive or physical performance within 1 month of study participation. Participants were instructed to refrain from caffeine for 12 h and from alcohol consumption and exercise training for 24 h and to arrive in an overnight‐fasted state before each experimental trial. Participants were instructed to maintain their usual exercise training frequency and volume throughout the study period. One participant withdrew after experiencing volitional exhaustion during one of the trials and was not part of the *n* = 16 data presented herein.

### 2.3 Experimental design

Using a single‐blind, randomized crossover design, participants visited the laboratory on three separate occasions over a 10‐ to 16‐day period, comprising one familiarization and two main experimental trials separated by a washout period of >48 h. During the first visit, participants were familiarized with the protocol, equipment, weighted ruck, subjective measurement scales and all tests of cognitive performance, in order to reduce the likelihood of a learning effect. Dietary intake data (24 h recall) were collected and analysed using the automated self‐administered 24 h (ASA24) dietary assessment tool (version 2021), developed by the National Cancer Institute, Bethesda, MD, USA, to ensure that participants were not currently consuming a ketogenic diet.

**FIGURE 1:**
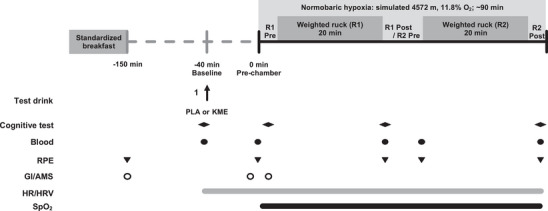
Schematic diagram of the experiment to investigate whether acute ingestion of (*R*)‐3‐hydroxybutyl (*R*)‐3‐hydroxybutyrate (R‐BD R‐βHB) ketone monoester (KME) impacts cognitive performance and oxygen saturation ($S_{pO_{2}}$) at rest and during moderate‐ to vigorous‐intensity exercise in the form of a weighted treadmill ruck during rapid‐onset acute hypoxic exposure. Abbreviations: GI/AMS, Gastrointestinal & Acute Mountain Sickness Questionnaire; HR/HRV, Heart Rate and Heart Rate Variability; KME, Ketone Monoester; PLA, Placebo; R1, Ruck 1; R2, Ruck 2; RPE, Rated Perceived Exertion; SpO2, Oxygen Satuation.

The two main experimental trials (visits 2 and 3) were composed of an exercise protocol consisting of 2 × 20 min weighted rucks (∼22 kg) at 3.2 km/h on a 10% incline, with the battery of cognitive performance tests administered before, within and immediately after exercise during hypoxic exposure (Figure 1). The primary outcome was cognitive performance between KME and a calorie‐ and taste‐matched placebo (PLA) condition, with secondary outcomes including $S_{pO_{2}}$, circulating βHB, glucose and lactate concentrations, heart rate (HR), parameters of heart rate variability (HRV), rating of perceived exertion (RPE), symptoms of AMS and symptoms of gastrointestinal (GI) disturbances.

### 2.4 Assessments of cognitive performance

At baseline, pre‐exercise (R1 Pre), after the first ruck (R1 Post) and after the second ruck (R2 Post), cognitive performance, including processing speed, selective attention, interference and executive functioning, was assessed using a self‐directed computerized Automated Neuropsychological Assessment Metric (ANAM^R^) test with established test–retest reliability for the Stroop color and word task (ANAM‐4, Vista Life Sciences, USA), a self‐directed tablet‐based Defense Automated Neurobehavioral Assessment (DANA; AnthroTronix Inc., USA) for the code substitution task, and a shoot simulation task (VirTra Inc., USA). All tests were performed in a sound‐insulated room in controlled conditions (i.e., appropriate lighting, as quiet as possible, and isolation from unnecessary stimuli and timing feedback). Participants were instructed to complete the battery of tests as quickly and accurately as possible.

#### 2.4.1 Stroop colour and word task

The Stroop color and word task measures cognitive flexibility, processing speed and executive function (Periáñez et al., 2021) and consists of three ‘blocks’. In the first block (‘neutral’), the words RED, GREEN and BLUE are presented individually in black text on the display. The user is instructed to read each word aloud and to press a corresponding key for each word (1 for RED, 2 for GREEN, 3 for BLUE). In the second block (‘congruent’), a series of XXXXs is presented on the display in one of three colours (red, green or blue). The user is instructed to say the colour of the XXXXs aloud and press the corresponding key based on colour. In the third block (“incongruent”), a series of individual words (RED, GREEN and BLUE) are presented in a colour that does not match the name of the colour depicted by the word. The user is instructed to press the response key assigned to that colour. Outcome measures are correct answers (number and percentage), reaction time (RT; in milliseconds), reaction time for correct responses (CRT) and the interference score.

#### 2.4.2 Code substitution task

The code substitution task was performed in the form of a simultaneous (CSS) and a delayed (CSD) code substitution task at each time point. For CSS, the participant refers to a code of nine symbol–digit pairs displayed across the upper portion of the screen. A sequence of single symbol–digit pairs is shown below the key, and the participant indicates whether or not the single pair matches the code by pressing ‘Yes’ or ‘No’. For CSD, the nine symbol–digit pairs are not displayed on the screen. However, the participant is still shown a sequence of single symbol–digit pairs and is asked to recall from memory whether these are the correct pairs from the CSS test that just occurred, with, importantly, the symbol–digit pairs changing at each time point. Outcome measures in both CSS and CSD are cognitive efficiency (CE; correct answers per minute), incorrect scores, RT, and RT for correct responses.

#### 2.4.3 Shoot simulation task

The shoot simulation task was a vigilance task with a real M4 rifle modified with a laser pin and CO_2_ cartridge (Malecek et al., 2023) (VirTra Inc., AZ, USA). Participants shot at a projector screen at a set distance displaying targets. At each time point (Baseline, R1 Post and R2 Post), participants were shown three different targets described as ‘shoots’ (all bullseyes of different colours, six in total), and were instructed to shoot only these targets as fast and as accurately as possible. A total of 30 targets were shown during each time point, one at a time for 1.5 s (15 ‘shoots’ and 15 ‘no shoots’) to ensure accurate shot capture via internal piloting. Outcome measures are a total score based on where the hit is relative to the bullseye (in a 1, 2, 3 scoring system, in which a higher score indicates a more accurate shot), RT, and numbers of hits, misses and incorrect target hits.

### 2.5 Main experimental trials

The main experimental trials were performed in a single‐blind, placebo‐controlled, randomized crossover design. Visits 2 and 3 were identical in terms of pre‐test preparation (standardized physical activity and instructed to replicate diet for 24 h before each visit), the exercise challenge and the test battery. The visits differed only in the drinks consumed before undergoing exercise and cognitive testing during hypoxia (i.e., KME vs. PLA).

Participants arrived in the morning (07.00–08.00 h) in a fasted state, completed a behavioural compliance questionnaire, provided a urine sample for urine specific gravity, and were weighed to the nearest 0.05 kg using calibrated digital weighing scales. After a resting (baseline) blood sample was collected, participants were fed a standardized breakfast consisting of a bagel (Dave's Killer Bread, OR, USA; 260 kcal, 3 g fat, 48 g carbohydrate and 11 g protein) with a single serving of peanut butter (J.M. Smuckers Co.; 250 kcal, 21 g fat, 11 g carbohydrate and 9 g protein) to replicate the fed state. After consumption, participants rested for ∼2 h, during which they completed the baseline assessments of cognitive performance, before ingesting the respective test drinks for KME or PLA ∼40 min prior to the onset of hypoxic exposure. The KME condition consisted of 573 mg/kg body mass of R‐BD R‐βHB KME (120 kcal/25 g; Pure Δ G ketone monoester; HVMN Inc., USA). This commercially available KME was mixed directly with a carbohydrate solution providing 40 g dextrose. The PLA beverage was a ketone‐free, isocaloric, taste‐matched placebo that contained 668 mg/kg body mass of maltodextrin combined with 40 g dextrose. The PLA drink was taste‐matched using a bitterness additive (Bittrex, USA) and internally piloted to ensure participant blinding. Both drinks were dissolved in water to 500 mL and given to participants in an opaque sports bottle. All dry ingredients were measured to the nearest 0.001 g on a calibrated balance scale (Denver Instruments, Bohemia, NY, USA). Participants were given 20 mL of calorie‐free flavoured drink mix (MIO, Kraft Heinz, Chicago, IL, USA) immediately after ingesting the experimental drinks to remove any lingering taste.

Approximately 40 min after ingestion, participants entered the normobaric altitude‐simulation chamber (Hypoxico, New York, USA; 4572 m, 11.8% O_2_) and rested in a seated position for 5 min before completing the pre‐exercise cognitive performance assessments. Then participants completed the 2 × 20 min standardized weighted rucks (MOLLE II, USA; ∼22 kg, 3.2 km/h, 10% incline) on a Woodway 4front treadmill (Woodway Inc., USA). Prior to implementation, the protocol was piloted (*n* = 6) for feasibility (ruck weight and exercise tolerance), safety, and indices of cardiorespiratory demand ($S_{pO_{2}}$ and HR). Subjective measures were collected in the final minute of each ruck. Participants remained in the altitude chamber until the R2 Post time point at which cognitive performance was assessed, which resulted in ∼90 min total exposure time.

### 2.6 Blood O_2_ saturation, HR and HRV

Heart rate and HRV were measured continuously throughout the trials (V800 Polar, Polar, Kempele, Finland). Once participants entered the chamber, $S_{pO_{2}}$ was measured with the Nellcor bedside respiratory patient monitoring system (Covidien, Dublin, Ireland) placed upon the forehead over the supraorbital artery. Before entering the chamber and during the recovery period outside, systemic oxygen saturation ($S_{pO_{2}}$) was measured by pulse oximetry with the Nellcor bedside respiratory patient monitoring system (Covidien).

### 2.7 Blood sample analysis

Concentrations of blood R‐βHB (Precision Xtra, Abbott Diabetes Care, USA), glucose (Precision Xtra) and lactate (Lactate Plus, Nova Biomedical, USA) were measured in finger stick capillary samples using commercially available hand‐held devices. Blood samples were collected using a lancet after alcohol cleaning. The first droplet was wiped away with a cotton swab, and subsequent droplets were used for analysis.

### 2.8 Subjective measures of exertion, symptoms of GI disturbances and AMS

Subjective measures of exertion, including RPE, leg discomfort scale, breathlessness intensity scale, anxiety of breathing, and anxiety of leg discomfort were recorded on a Likert scale (1–10; from 1, none to 10, maximal) (Faull et al., 2019). Likewise, participants were asked to rate GI symptoms (heartburn, bloating, nausea, vomiting, intestinal cramps, abdominal pain, flatulence and diarrhoea) and symptoms of AMS (dizziness, headache, muscle cramp and urge to urinate) on a Likert scale (0–8; from 0, no symptoms to 8, unbearable symptoms) (Stubbs et al., 2019).

### 2.9 Statistical analysis

All statistical analyses and graphical representation of data were performed using Prism v.9 (GraphPad Software, San Diego, CA, USA). The normality of data was assessed with the Shapiro–Wilk normality test, which all data passed. Data are presented as the mean ± SD, or the mean difference (lower, higher 95% confidence limits of mean) where indicated. A two‐way (time × condition) repeated‐measures ANOVA was used to identify differences, if any, between the conditions across time. When a time × condition interaction effect or a main effect of condition was observed, *post hoc* pairwise comparisons were performed with the Sidak correction applied, and multiplicity‐adjusted *P*‐values are reported. Sphericity was not assumed, and the Greenhouse–Geiser correction was applied to all ANOVAs. The threshold for statistical significance was set at *P* ≤ 0.05 for all tests.

## 3 RESULTS

### 3.1 Blood metabolites

**FIGURE 2:**
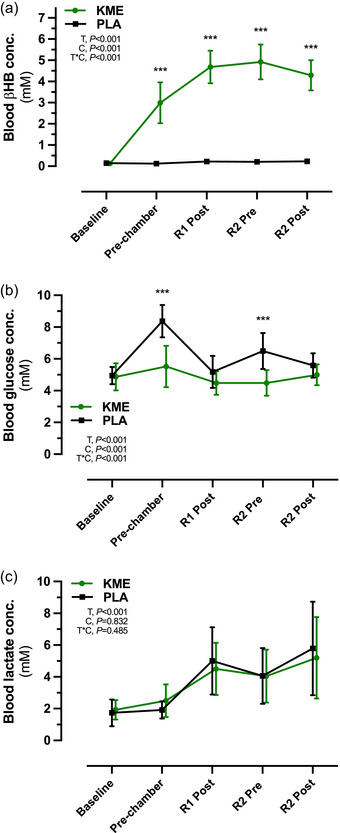
Blood R‐βHB (a), glucose (b) and lactate (c) concentrations during ketone monoester (KME) and placebo (PLA) conditions at Baseline, Pre‐chamber, after ruck 1 (R1 Post) and before and after ruck 2 (R2 Pre and R2 Post, respectively). Data are presented as the mean ± SD. Abbreviations: C, condition (KME vs. PLA); PLA, placebo; R‐βHB, (*R*)‐3‐hydroxybutyrate; T, time; T*C, time × condition interaction. ***P < 0.001 for KME versus PLA.

Fasting concentrations of βHB (KME, 0.12 ± 0.05 mM; PLA, 0.14 ± 0.06 mM), glucose (KME, 87.6 ± 15.3 mg/dL; PLA, 89.1 ± 9.7 mg/dL) and lactate (KME, 1.92 ± 0.61 mM; PLA, 1.73 ± 0.84 mM) did not differ between trials at baseline (Figure 2). A main effect of time and condition (both *P *< 0.001) and a time × condition interaction effect (*P *< 0.001) were observed for plasma R‐βHB concentrations. Blood R‐βHB was significantly elevated at all subsequent time points after consuming the KME compared with PLA and peaked prior to R2 (*P *< 0.001). Ingestion of KME resulted in a rise in circulating R‐βHB concentrations (2.99 ± 0.96 mM, *P *< 0.001) by the start of the first ruck (R1). The R‐βHB concentrations continued to rise and peaked at 4.93 ± 0.82 mM prior to starting the second ruck (R2), and they remained elevated at >4 mM for the remaining KME trial (*P *< 0.001) (Figure 2a). A main effect of time and condition (both *P *< 0.001) and a time × condition interaction effect (*P *< 0.001) were observed for plasma glucose concentrations. Blood glucose was significantly lower in the KME condition compared with PLA by 30 min postingestion and before R2 (both *P *< 0.001) (Figure 2b). Blood lactate concentration was not significantly different between conditions at any time point, although lactate concentrations increased throughout the experimental trials (main effect of time, *P *< 0.001), such that values in both conditions at R1 Post, R2 Pre and R2 Post were elevated above Baseline and Pre‐chamber (Figure 2c).

### 3.2 Cognitive performance outcomes

**FIGURE 3:**
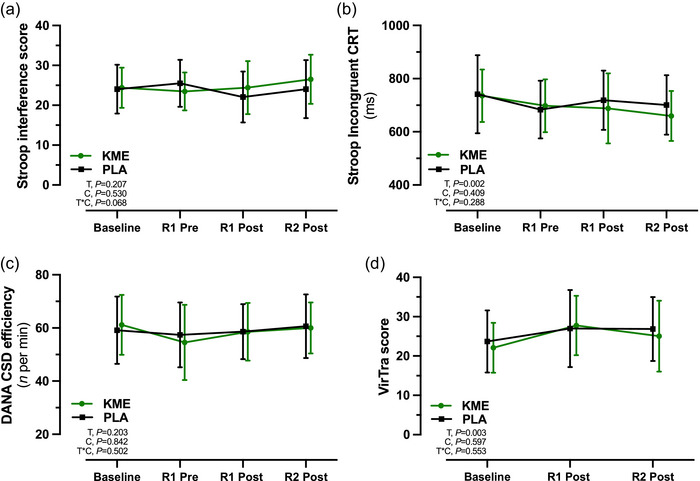
Cognitive performance assessed by Stroop interference score (a), Stroop incongruent correct reaction time (CRT) (b) and DANA code substitution delayed efficiency (c) during ketone monoester (KME) and placebo (PLA) conditions at Baseline, before ruck 1 (R1 Pre), after ruck 1 (R1 Post) and after ruck 2 (R2 Post). (d) The VirTra shoot simulation occurred only at Baseline, R1 Post and Post R2. Data are presented as the mean ± SD. Abbreviations: C, condition (KME vs. PLA); T, time; T*C, time × condition interaction.

All cognitive performance outcomes did not differ at baseline between conditions (Figure 3; Table 1). A main effect of time was observed for code substitution task CSS correct RT, CE and incorrect responses (*P* = 0.01, *P* = 0.04 and *P* = 0.03, respectively), code substitution task CSD incorrect responses (*P* = 0.03), Stroop color and word task neutral and incongruent CRT (*P *< 0.001 and *P* = 0.002, respectively), and neutral and incongruent percentage correct (*P* = 0.001 and *P* = 0.006, respectively) (Table 1). The total score in the shoot simulation task increased during the trials (main effect of time, *P* = 0.003) (Figure 3; Table 1). The absence of time × condition interaction effects across all outcome measures indicates no differences between KME and PLA on cognitive performance for outcomes reported in Figure 3 and Table 1, with similar findings also evident for the Stroop color and word task for %correct neutral, %correct congruent, correct RT neutral, correct RT congruent, CSS incorrect, CSD incorrect, shoot simulation hit and shoot simulation miss (data not shown).

**TABLE 1:** Cognitive performance outcomes.

	Time point				
Parameter	Baseline	R1 Pre	R1 Post	R2 Post	*P*‐value
**Stroop incongruent correct (%)**					
PLA KME	93.9 ± 5.1 93.5 ± 5.3	91.2 ± 4.6 92.6 ± 4.2	89.5 ± 6.0 90.3 ± 4.0	91.1 ± 4.7 91.7 ± 4.9	Time, *P *= 0.006 Condition, *P *= 0.471 Interaction, *P *= 0.660
**Stroop incongruent correct RT (ms)**					
PLA KME	741.7 ± 146.9 735.7 ± 98.4	683.5 ± 108.4 698.2 ± 99.6	718.9 ± 111.2 688.0 ± 131.5	700.7 ± 112.3 659.6 ± 94.1	Time, *P *= 0.002 Condition, *P *= 0.409 Interaction, *P *= 0.288
**Stroop interference score**					
PLA KME	24.05 ± 6.1 23.3 ± 6.5	25.51 ± 5.8 23.45 ± 4.7	22.06 ± 6.3 24.44 ± 6.6	24.03 ± 7.2 26.52 ± 6.1	Time, *P *= 0.19 Condition, *P *= 0.63 Interaction, *P *= 0.06
**DANA CSD correct RT (ms)**					
PLA KME	969.8 ± 241.2 994.6 ± 169.5	978.7 ± 245.0 1008.3 ± 276.7	985.6 ± 152.9 932.0 ± 200.7	933.1 ± 205.6 948.6 ± 118.7	Time, *P *= 0.26 Condition, *P *= 0.68 Interaction, *P *= 0.54
**DANA CSD efficiency (correct response/min)**					
PLA KME	57.3 ± 14.1 60.6 ± 11.0	56.1 ± 12.8 54.3 ± 13.6	56.6 ± 12.7 55.7 ± 15.2	60.0 ± 11.7 60.1 ± 9.2	Time, *P *= 0.20 Condition, *P *= 0.84 Interaction, *P *= 0.50
**DANA CSS correct RT (ms)**					
PLA KME	1146.4 ± 197.4 1186.1 ± 307.8	1117.2 ± 171.4 1134.0 ± 197.2	1140.2 ± 201.6 1118.2 ± 222.7	1055.7 ± 156.7 1074.8 ± 176.1	Time, *P *= 0.01 Condition, *P *= 0.71 Interaction, *P *= 0.53
**DANA CSS efficiency (correct response/min)**					
PLA KME	53.05 ± 9.0 52.63 ± 12.5	53.30 ± 7.2 52.93 ± 8.9	52.71 ± 8.7 53.99 ± 10.0	56.18 ± 7.2 55.35 ± 8.6	Time. *P =* 0.04 Condition, *P *= 0.96 Interaction, *P *= 0.72
**VirTra correct RT (ms)**					
PLA	1.29 ± 0.07	–	1.27 ± 0.07	1.30 ± 0.05	Time, *P *= 0.08
KME	1.29 ± 0.07	–	1.25 ± 0.06	1.27 ± 0.07	Condition, *P *= 0.26 Interaction, *P *= 0.19
**VirTra correct RT (#)**					
PLA	23.68 ± 7.9	–	27.0 ± 9.8	26.87 ± 8.1	Time, *P *= 0.003
KME	22.12 ± 6.3	–	27.75 ± 7.5	25.06 ± 9.0	Condition, *P *= 0.59 Interaction, *P *= 0.55

*Note*: Data are presented as the mean ± SD, *n* = 16. Baseline was taken as −150 min before the start of exercise; see Figure 1 for more details.

Abbreviations: CSD, Code Substitution Delayed; CSS, Code Substitution Simultaneous; DANA, Defense Automated Neurobehavioral Assessment; KME, ketone monoester; PLA, placebo; RT, reaction time.

### 3.3 Oxygen saturation

**FIGURE 4:**
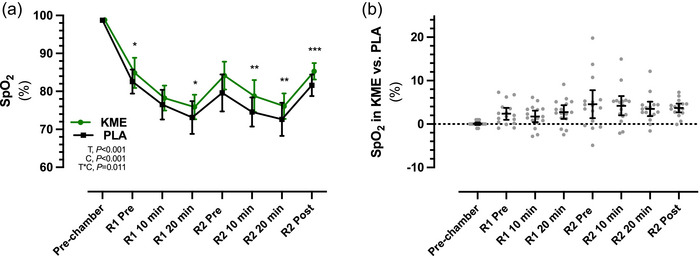
Average oxygen saturation ($S_{pO_{2}}$) (a), and mean differences in $S_{pO_{2}}$ between trials (b), during ketone monoester (KME) and placebo (PLA) conditions at Pre‐chamber, and before (Pre) and during (10 and 20 min) ruck 1 (R1) and ruck 2 (R2). Data are presented as the mean ± SD in (a) and as the mean ± 95% confidence limits in (b). Abbreviations: C, condition (KME vs. PLA); T, time; T*C, time × condition interaction. *P < 0.05, **P < 0.01 and ***P < 0.001 for KME versus PLA.

Before entering the altitude chamber, there were no differences in $S_{pO_{2}}$ between conditions (KME, 98.80 ± 0.40%; PLA, 98.75 ± 0.44%). During high‐altitude exposure, the decline in $S_{pO_{2}}$ was attenuated in KME during the entire exposure time compared with PLA (main effect of condition, *P *< 0.001; Figure 4a). Pairwise comparisons revealed higher %$S_{pO_{2}}$ at R1 Pre [2.4% (0.3, 4.4); *P* = 0.020], R1 20 min [2.8% (0.4, 5.1); *P* = 0.016], R2 10 min [4.2% (0.9, 7.5); *P* = 0.008], R2 20 min [3.5% (1.0, 5.9); *P* = 0.003] and R2 Post [3.6% (2.2, 5.2); *P *< 0.001; Figure 4b].

### 3.4 Heart rate and HRV parameters

**FIGURE 5:**
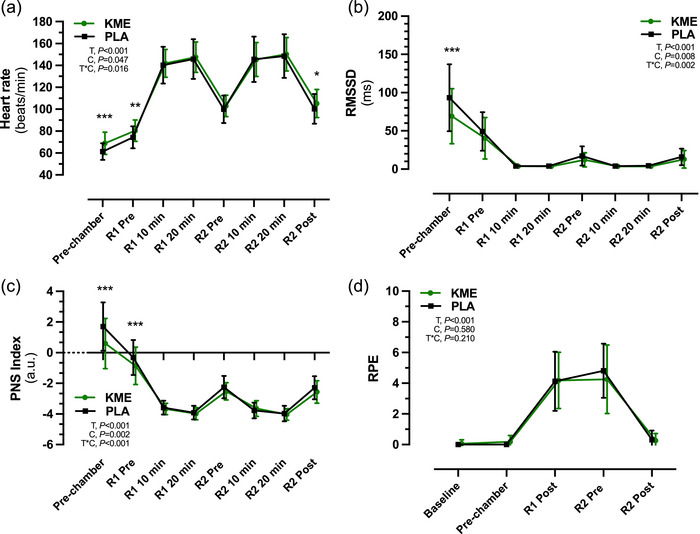
Heart rate (HR) (a), room mean square of successive differences (RMSSD) (b) and parasympathetic (PNS) index (c) during ketone monoester (KME) and placebo (PLA) conditions Pre‐chamber and before (Pre) and during (10 and 20 min) ruck 1 (R1) and ruck 2 (R2). (d) Rating of perceived exertion (RPE) was taken at Baseline, Pre‐chamber, after ruck 1 (R1 Post) and before and after ruck 2 (R2 Pre and R2 Post, respectively). Data are presented as the mean ± SD. Abbreviations: C, condition (KME vs. PLA); T, time; T*C, time × condition interaction. *P < 0.05, **P < 0.01 and ***P < 0.001 for KME versus PLA.

A main effect for time was observed for the root mean square of successive differences (RMSSD), R–R interval, stress index, sympathetic nervous system (SNS) and parasympathetic nervous system (PNS) indexes (all *P *< 0.001; Table 2). Condition × time interaction effects were observed in RMSSD (*P* = 0.002) and R–R interval (*P *< 0.001), indicating a difference in autonomic function between the KME and PLA conditions. A main effect of time (*P *< 0.001) and condition (*P* = 0.047) and a time × condition interaction effect (*P* = 0.016) were observed for HR. Average HR was higher in KME compared with PLA before (Pre‐chamber) and after (R1 Pre) hypoxic exposure and after completion of R2 (R2 Post), but did not differ between conditions (Figure 5a). As a result, the R–R interval was greater in KME compared with PLA at the same time points (*P *< 0.001; Table 2). The RMSSD was significantly lower in KME compared with PLA [−23.7 ms (−35.7, −11.7); *P *< 0.001] 30 min after ingestion of the drinks (Pre‐chamber), but not different at any other time points (Figure 5b). The PNS index was significantly lower in KME compared with PLA before (Pre‐chamber) and after (R1 Pre) hypoxic exposure (*P *< 0.001 and *P* = 0.005, respectively), whereas the SNS index did not differ between conditions at any time point (Table 2), and likewise, no other HR parameters measured were different between conditions [e.g., low‐frequency, high‐frequency and total power (data not shown)]. The RPE increased during the respective trials but did not differ between conditions (Figure 5d)

**TABLE 2:** Heart rate variability outcomes.

	Time point								
Parameter	Pre‐chamber	R1 Pre	R1 10 min	R1 20 min	R2 Pre	R2 10 min	R2 20 min	R2 Post	*P*‐value
**R–R interval (ms)**									
PLA KME	991.9 ± 126.4 889.9 ± 144.9***	820.6 ± 112.1 756.8 ± 99.3***	433.3 ± 51.54 426.5 ± 41.6	417.6 ± 53.6 410.2 ± 40.6	609.7 ± 77.0 587.2 ± 56.0	420.2 ± 60.9 416.9 ± 45.8	411.1 ± 56.8 403.0 ± 41.3	608.0 ± 81.4 578.8 ± 71.7	Time, *P *< 0.001 Condition, *P *< 0.001 Interaction, *P *< 0.001
**RMSSD (ms)**									
PLA KME	93.2 ± 43.8 69.9 ± 37.1***	49.1 ± 25.1 40.1 ± 28.1***	3.8 ± 2.3 3.8 ± 2.3	3.9 ± 2.7 3.6 ± 1.7	17.0 ± 12.5 22.2 ± 3.2	4.0 ± 2.3 3.7 ± 1.8	4.2 ± 2.9 3.6 ± 1.8	16.0 ± 10.8 12.4 ± 11.9	Time, *P *< 0.001 Condition, *P =* 0.009 Interaction, *P *= 0.002
**Stress index (a.u.)**									
PLA KME	5.4 ± 2.3 7.0 ± 3.0	8.0 ± 2.9 10.5 ± 4.0	60.1 ± 30.2 60.9 ± 27.4	71.8 ± 25.8 81.3 ± 26.0	18.0 ± 7.1 19.0 ± 7.0	57.7 ± 28.9 62.5 ± 28.6	71.9 ± 28.2 82.5 ± 20.9	18.8 ± 8.9 21.7 ± 9.5	Time, *P < *0.001 Condition, *P *= 0.043 Interaction, *P *= 0.213
**SNS index (a.u.)**									
PLA KME	−0.89 ± 0.8 −0.15 ± 1.1	0.39 ± 1.0 1.19 ± 1.2	14.37 ± 6.1 14.69 ± 4.0	17.73 ± 6.3 19.73 ± 5.9	3.78 ± 2.0 4.17 ± 1.8	14.59 ± 6.3 14.76 ± 5.3	18.10 ± 6.9 20.17 ± 5.3	3.97 ± 2.4 4.81 ± 2.5	Time, *P < *0.001 Condition, *P *= 0.09 Interaction, *P *= 0.58
**PNS index (a.u.)**									
PLA KME	1.70 ± 1.5 0.59 ± 1.6***	−0.32 ± 1.1 −0.86 ± 1.2**	−3.58 ± 0.4 −3.67 ± 0.3	−3.91 ± 0.4 −3.97 ± 0.3	−2.24 ± 0.7 −2.52 ± 0.5	−3.77 ± 0.5 −3.79 ± 0.4	−3.97 ± 0.5 −4.02 ± 0.3	−2.29 ± 0.7 −2.56 ± 0.7	Time, *P < *0.001 Condition, *P *= 0.54 Interaction, *P *= 0.87

*Note*: Data are presented as the mean ± SD, *n* = 16.

Abbreviations: a.u., arbitrary units; KME, ketone monoester; PLA, placebo; PNS, parasympathetic nervous system; RMSSD, root mean square of successive differences between normal heartbeats; SNS, sympathetic nervous system.

***P < 0.001, **P < 0.01 for KME versus PLA.

### 3.5 Symptoms of GI disturbance and AMS

Heartburn, bloating, nausea, vomiting, intestinal cramps, abdominal pain, flatulence, diarrhoea, dizziness, headache and muscle cramps remained absent or very mild across both treatments during the acute hypoxic exposure (Table S1).

### 3.6 Assessment of blinding

After the experimental protocol in the third visit was completed, but before participants were informed of their results achieved during each trial, participants were asked to indicate which trial they believed to be KME and PLA. Participants answered correctly six times, incorrectly eight times, and ‘I don't know’ twice, which suggests successful blinding, in that participants were unable to detect treatment versus placebo outside of random chance.

## 4 DISCUSSION

We investigated whether acute ingestion of an exogenous ketone supplement in the form of R‐BD R‐βHB KME could acutely impact $S_{pO_{2}}$ and cognitive performance during acute, short‐duration acute hypoxic exposure at rest and during moderate‐intensity exercise. Ingestion of R‐BD R‐βHB KME resulted in circulating R‐βHB concentrations of >3 mM through the ∼90 min of hypoxic exposure and resulted in an attenuation of the decline in $S_{pO_{2}}$ of 2.4% in R1 and 4.2% in R2. However, this $S_{pO_{2}}$ ‘advantage’ compared with PLA did not translate into differences in the primary outcome of cognitive performance during the experimental protocol.

Ingestion of 573 mg/kg of R‐BD R‐βHB KME in a postprandial state rapidly increased circulating R‐βHB concentrations to ∼3 mM at 20 min after ingestion, and concentrations remained elevated at >4 mM on average throughout the hypoxic exposure. These circulating R‐βHB concentrations align with previous work using the same single dose of KME before exercise (Cox et al., 2016; Dearlove et al., 2021). Blood glucose concentrations were lower throughout KME compared with PLA, which is consistent with prior literature on the effect of acute nutritional ketosis to attenuate the rise in postprandial blood glucose concentration (Falkenhain et al., 2022). However, it is important to note that in the present study, additional carbohydrate (∼50 g) was consumed during PLA in order for both conditions to be isocaloric, which differs from our previous work on hypoxia that compared KME with a non‐caloric PLA (Coleman et al., 2021). Given the higher dose of carbohydrate in PLA compared with KME, the differences in blood glucose concentration observed between conditions are likely to be attributable, in large part, to the higher carbohydrate dose ingested. The addition of carbohydrate is likely to have influenced the biological impact of exogenous ketones, because prior studies have demonstrated that co‐administration of KME with carbohydrate alters the oxygen cost and substrate utilization during exercise compared with KME alone (Brady & Egan, 2024; Cox et al., 2016). Blood lactate concentrations did not differ between trials before or after exercise. This finding contradicts the previous finding of attenuated lactate response to exercise during hypoxia after KME ingestion (Poffé, Robberechts et al., 2021), but might reflect differences between the studies in the exercise type, intensity and duration of the respective studies. The intensity of exercise in the present study was markedly lower than that in a previous study, which consisted of varied‐intensity cycling exercise at 60%–90% of the power output at lactate threshold, a 15 min time trial, and an all‐out sprint at 175% of the power output at lactate threshold (Poffé, Robberechts et al., 2021). In general, there remains no consistent pattern in whether KME ingestion attenuates the increase in blood lactate concentrations in response to moderate to high‐intensity exercise (Evans et al., 2022).

The present observation of an attenuation of the decline in $S_{pO_{2}}$ of 2.4%–4.2% during simulated high‐altitude exposure aligns with previous investigations that used acute nutritional ketosis as a countermeasure during severe hypoxic exposure at rest or during exercise (Coleman et al., 2021; Poffé, Robberechts et al., 2021). The proposed mechanism for attenuation of the decline in $S_{pO_{2}}$ is the increased hypoxic ventilatory response in response to declines in pH after R‐BD R‐βHB KME ingestion. Although not measured in the present study, multiple studies have observed a decrease in pH of ∼0.5 units during acute nutritional ketosis in normoxia (Dearlove et al., 2021; Poffé, Ramaekers et al., 2021; Poffé, Wyns et al., 2021) and hypoxia (Poffé, Robberechts et al., 2021). An increased ventilation rate, leading to increased breathing frequency and tidal volume, has also been observed (Dearlove et al., 2019; McCarthy et al., 2023; Poffé, Robberechts et al., 2021). Importantly, co‐ingestion of R‐BD R‐βHB KME with an acid buffer in the form of sodium bicarbonate reduced the effect on attenuating the decline in $S_{pO_{2}}$, and increased ventilation during hypoxic exposure (Poffé, Robberechts et al., 2021), which suggests that the increased rate of ventilation is indeed a mechanism involved in attenuation of declines in $S_{pO_{2}}$ in hypoxic environments. In the present study, the difference in $S_{pO_{2}}$ during KME compared with PLA increased from 2.4% to 4.2% from Ruck 1 to Ruck 2, which suggests increased resiliency to the decline in $S_{pO_{2}}$ with prolonged hypoxic exposure during acute nutritional ketosis. However, these differences in $S_{pO_{2}}$ did not translate into differences in cognitive performance during the experimental protocol before or after exercise compared with PLA.

The rationale for why acute nutritional ketosis might produce enhanced resilience to maintain cognitive performance during hypoxic exposure is proposed as a combination of greater O_2_ availability (as observed) and effects on circulating brain‐derived neurotrophic factor (Walsh et al., 2020) and cerebral blood flow (Walsh et al., 2021). Moreover, ketone bodies provide the brain with a complimentary or alternative substrate to glucose, which is potentially of utility in hypoxic environments, given that carbohydrate utilization might decrease in response to hypoxia‐induced insulin resistance (Myette‐Côté et al., 2018; Pasiakos et al., 2021). In two previous studies by our group (each using a randomized, crossover, placebo‐controlled design matching the design implemented here), we tested the potential effects of KME on hypoxia‐induced declines in DANA‐assessed cognitive performance in the non‐exercising, resting state. In both trials, we noted some attenuation of cognitive decline after ingesting KME: (1) in code substitution tasks at rest at simulated 5029 m (Coleman et al., 2021); and (2) in code substitution tasks at rest at simulated 6096 m (McClure et al., 2024; accompanying submitted manuscript). In the more severe hypoxic conditions (McClure et al., 2024; accompanying submitted manuscript), the apparent cognitive performance benefits of KME were coupled with a 4.3% $S_{pO_{2}}$ advantage, which we suggest to have played a role in the resilience of cognitive performance.

In the present study, no cognitive advantage of KME was noted, most probably owing to the co‐intervention with two bouts of moderate to vigorous exercise. Acute exercise can temporarily enhance cognitive performance (Chang et al., 2012), which could have the effect of superseding any potential benefits to cognitive performance induced by KME during hypoxic exposure. Other design factors potentially contributing to the lack of cognitive benefit in this exercise trial versus the two prior resting‐state trials include the severity of hypoxic exposure (4572 vs. 5029 and 6096 m) and the duration of hypoxic exposure time at rest before cognitive testing, because the time of useful consciousness varies with the severity and duration of hypoxic exposure (Shaw et al., 2021). Acute hypoxic exposure impairs cognitive performance (Shaw et al., 2021), which we also confirmed at rest in our prior studies (Coleman et al., 2021; McClure et al., 2024; accompanying submitted manuscript). Conversely, not all measures of cognitive performance declined during the hypoxic exposure in either PLA or KME in this study, which we attribute to the potent effect of acute exercise improving cognitive performance being greater than the declines expected under hypoxia. Skeletal muscle and cerebral oxygenation were not measured; thus, despite the effects found on blood $S_{pO_{2}}$, further investigation would be required to determine whether oxygenation of specific tissues is influenced in these experimental conditions.

A new finding in this study is the effect of KME in altering HRV at rest, with and without the hypoxic exposure. Hypoxic exposure at similar simulated altitudes has been shown to alter HRV at rest and during exercise by increasing heart rate and lowering parasympathetic cardiac activation (Buchheit et al., 2004; Rupp et al., 2013). KME elicited a parasympathetic withdrawal in both normoxia and hypoxia when at rest, resulting in an elevated heart rate during rest (∼8 beats/min) compared with PLA. Interestingly, once exercise commenced during the hypoxic exposure, there were no differences in autonomic function or heart rate. Ingestion of KME affected HRV further by decreasing mean R–R intervals and RMSSD at rest compared with PLA, leading to an elevated heart rate and lower parasympathetic cardiac activation prior to exercise in the hypoxic environment. However, the addition of exercise to the hypoxic exposure eliminated any differences in heart rate or HRV between trials. Analogously, even 30% of maximal voluntary contraction using a handgrip exercise abolished differences in parasympathetic/sympathetic tone induced by prolonged cold exposure, confirming the hierarchical impact of exercise on regulating autonomic function (Sanchez‐Gonzalez & Figueroa, 2013). These data are consistent with previous findings on heart rate during exercise of varying intensities during hypoxic exposure, with KME ingestion being no different in comparison to PLA (Poffé, Wyns et al., 2021). However, the effects of KME ingestion on HRV at rest and during exercise in normal and hypoxic conditions might warrant future investigation, because the mechanisms underlying these relationships remain to be understood fully and are in contrast to findings showing increases in mean R–R and parasympathetic cardiac activation after adherence to a ketogenic diet (Polito et al., 2022).

There are a few limitations in the present study. First, this experimental model of acute hypoxic exposure presents a rapid (<1 h) onset and does not include a hypobaric stimulus. The absence of AMS‐related symptoms, even with the acute exposure to severe hypoxia used, limits the study findings to a very specific situation and context. The results might not apply or be relevant in ‘typical’ gradual exposures to altitude over several days, including recommended acclimatization procedures. Second, although we observed differences in oxygen saturation and in metabolic, autonomic and HR responses in healthy males exercising during acute hypoxia with KME administration, whether these results will translate to females or to individuals who are less active is not known. Third, capillary blood samples were analysed with point‐of‐care devices and might not accurately reflect venous circulation values by laboratory‐based measures, often by over‐estimating the R‐βHB concentration (Evans et al., 2022). Collection of blood gases would be necessary in order to confirm our hypothesized mechanism of action involving acidosis and declining pH. Lastly, while we assessed normobaric hypoxia, hypobaric hypoxia might also result in physiological differences, which are not possible to assess or infer from the present design (Coppel et al., 2015).

In conclusion, acute ingestion of KME had no impact on cognitive performance during exercise with acute hypoxic exposure despite attenuating the declines in systemic oxygen saturation compared with PLA. These results suggest that KME ingestion increased resilience to the $S_{pO_{2}}$ decline associated with acute hypoxic exposure during exercise, while cognitive performance and physical capacity were maintained. Acute ingestion of KME might therefore be a potential countermeasure for acute, rapid‐onset hypoxic exposure by attenuating declines in oxygen availability, without decrements to cognition or moderate exercise in hypoxic conditions. Further work could explore the ability of KME to maintain cerebral oxygenation and cerebral metabolic rate during other hypoxic exposures in both environmental (e.g., high‐altitude activities, such as mountaineering) and pathological conditions causing hypoxaemia, such as asthma, respiratory viral infections or chronic obstructive pulmonary disease.

## AUTHOR CONTRIBUTIONS

Conceived and designed research: Tyler S. McClure, Jeffrey Phillips and Andrew P. Koutnik; Performed experiments: Tyler S. McClure, Kody Coleman and Ed Chappe; Analysed data: Tyler S. McClure, Gary R. Cutter, Brendan Egan, Marcas M. Bamman and Andrew P. Koutnik; Interpreted results of experiments: Tyler S. McClure, Jeffrey Phillips, Dawn Kernagis, Gary R. Cutter, Brendan Egan, Marcas M. Bamman and Andrew P. Koutnik; Prepared figures: Tyler S. McClure, Brendan Egan and Andrew P. Koutnik; Drafted manuscript: Tyler S. McClure and Brendan Egan; Edited and revised manuscript: Tyler S. McClure, Jeffrey Phillips, Dawn Kernagis, Kody Coleman, Ed Chappe, Gary R. Cutter, Brendan Egan, Todd Norell, Brianna J. Stubbs, Marcas M. Bamman and Andrew P. Koutnik. All authors approved the final version of manuscript and agree to be accountable for all aspects of the work in ensuring that questions related to the accuracy or integrity of any part of the work are appropriately investigated and resolved. All persons designated as authors qualify for authorship, and all those who qualify for authorship are listed.

## CONFLICT OF INTEREST

HVMN Inc. had no involvement in data collection, analysis, or data interpretation. T.S.M., J.P., K.C., E.C., D.K., B.E. and M.M.B. declare no conflicts of interest and do not have any financial disclosures. B.J.S. has stock and stock options in companies that produce ketone products (HVMN Inc., Juvenescence Ltd, BHB Therapeutics Ltd and Selah Ltd) and is an inventor on patents that relate to ketone bodies. B.J.S. was an employee of HVMN Inc. at the time this study was conceived. G.R.C. participates on Data and Safety Monitoring Boards for Applied Therapeutics, AI therapeutics, AMO Pharma, Astra‐Zeneca, Avexis Pharmaceuticals, Bristol Meyers Squibb/Celgene, CSL Behring, Horizon Pharmaceuticals, Immunic, Karuna Therapeutics, Kezar Life Sciences, Mapi Pharmaceuticals Ltd, Merck, Mitsubishi Tanabe Pharma Holdings, Opko Biologics, Prothena Biosciences, Novartis, Regeneron, Sanofi‐Aventis, Reata Pharmaceuticals, Teva Pharmaceuticals, NHLBI (Protocol Review Committee), University of Texas Southwestern, University of Pennsylvania and Visioneering Technologies, Inc. G.R.C. participates as a consultant or Advisory Board member for Alexion, Antisense Therapeutics, Avotres, Biogen, Clene Nanomedicine, Clinical Trial Solutions LLC, Entelexo Biotherapeutics, Inc., Genzyme, Genentech, GW Pharmaceuticals, Hoya Corporation, Immunic, Immunosis Pty Ltd, Klein‐Buendel Inc., Linical, Merck/Serono, Novartis, Perception Neurosciences, Protalix Biotherapeutics, Regeneron, Roche and SAB Biotherapeutics. G.R.C. is President of Pythagoras Inc., a private consulting company located in Birmingham, AL, USA. A.P.K. is a patent inventor (US11452704B2 and US11596616B2) and is on the Scientific Advisory Board for Simply Good Foods and Nutrishus. Any opinions, findings and conclusions or recommendations expressed in this material are those of the authors and do not necessarily reflect the views of the United States Special Operations Command or the United States Department of Defense.

## Supporting information

Supplemental Table S1. Gastrointestinal and acute mountain sickness symptom questionnaire.

## Data Availability

The data that support the findings of this study are available upon request from the corresponding author.
