# Supplementary material for: Ketone monoester attenuates oxygen desaturation during weighted ruck exercise under acute hypoxic exposure but does not impact cognitive performance
Source: Exp Physiol. 2024 Aug 27;109(10):1768–81. doi: 10.1113/EP091789 (PMC11442785; doi:10.1113/EP091789)
Supplement: Supplementary file 1 — Supplemental Table S1. Gastrointestinal and acute mountain sickness symptom questionnaire. [file EPH-109-1768-s001.docx]

**Supplemental Table S1. Gastrointestinal and acute mountain sickness symptom questionnaire**

|  | **Time point** | | |
| --- | --- | --- | --- |
|  | **-150 min** | **30 min after ingestion** | **In chamber at rest** |
| **Heartburn** |  |  |  |
| PLA | 0±0.0 | 0±0.0 | 0±0.0 |
| KME | 0±0.0 | 0±0.2 | 0.06±0.2 |
| **Bloating** |  |  |  |
| PLA | 0±0.0 | 0±0.0 | 0±0.0 |
| KME | 0.06±0.2 | 0.06±0.2 | 0±0.0 |
| **Nausea** |  |  |  |
| PLA | 0.06±0.2 | 0.06±0.2 | 0±0.0 |
| KME | 0±0.0 | 0±0.0 | 0±0.0 |
| **Vomiting** |  |  |  |
| PLA | 0.12±0.5 | 0±0.0 | 0±0.0 |
| KME | 0±0.0 | 0.06±0.2 | 0.06±0.2 |
| **Intestinal Cramps** |  |  |  |
| PLA | 0±0.0 | 0±0.0 | 0±0.0 |
| KME | 0±0.0 | 0±0.0 | 0±0.0 |
| **Abdominal pain** |  |  |  |
| PLA | 0±0.0 | 0±0.0 | 0±0.0 |
| KME | 0±0.0 | 0±0.0 | 0±0.0 |
| **Flatulence** |  |  |  |
| PLA | 0.12±0.5 | 0.06±0.2 | 0±0.0 |
| KME | 0.18±0.7 | 0±0.0 | 0±0.0 |
| **Diarrhoea** |  |  |  |
| PLA | 0±0.0 | 0±0.0 | 0±0.0 |
| KME | 0±0.0 | 0±0.0 | 0±0.0 |
| **Dizziness** |  |  |  |
| PLA | 0±0.0 | 0±0.0 | 0.12±0.5 |
| KME | 0±0.0 | 0±0.0 | 0.12±0.3 |
| **Headache** |  |  |  |
| PLA | 0.06±0.2 | 0±0.0 | 0±0.0 |
| KME | 0±0.0 | 0±0.0 | 0.06±0.2 |
| **Muscle cramp** |  |  |  |
| PLA | 0.18±0.7 | 0.06±0.2 | 0.06±0.2 |
| KME | 0.06±0.2 | 0±0.0 | 0.06±0.2 |
| **Urge to urinate** |  |  |  |
| PLA | 0.18±0.5 | 0.12±0.3 | 0.3±0.6 |
| KME | 0±0.0 | 0.12±0.3 | 0.12±0.3 |
|  |  |  |  |

See Figure 1 for more details. Scores ranging from 0-8, 0: no symptoms; 8: unbearable symptoms. Data are presented as mean±SD, n=16
